# Exploring evidence use and capacity for health services management and planning in Swiss health administrations: A mixed-method interview study

**DOI:** 10.1371/journal.pone.0302864

**Published:** 2024-05-08

**Authors:** Aron Baumann, Kaspar Wyss

**Affiliations:** 1 Swiss Centre for International Health, Swiss Tropical and Public Health Institute, Allschwil, Switzerland; 2 University of Basel, Basel, Switzerland; University of Potsdam: Universitat Potsdam, GERMANY

## Abstract

**Background:**

Health administrations require evidence, meaning robust information, data, and research, on health services and systems. Little is known about the resources and processes available within administrations to support evidence-informed policymaking. This study assessed Swiss health administrations’ capacity for evidence use and investigated civil servants’ needs and perspectives regarding the role and use of evidence in health services management and planning.

**Methods:**

In this mixed-method study, we interviewed civil servants from Swiss German-speaking cantonal health administrations. We quantitatively assessed administrations’ organization-level capacity by applying six structured interviews using an existing measurement tool (ORACLe). Individual-level needs and perspectives regarding evidence use and capacity were qualitatively explored with twelve in-depth interviews that were analyzed using the framework method.

**Findings:**

Respondents indicated moderate evidence-use capacity in all administrations. Administrations displayed a similar pattern of high and low capacity in specific capacity areas, generally with considerable variation within administrations. Most administrations indicated high capacity for producing or commissioning evidence and close relationships with research. They showed limited capacity in the documentation of processes and availability of tools, programs, or training opportunities. Administrations place the responsibility for engagement with evidence at the level of individual civil servants rather than at the organizational level. Although administrations highly value evidence-informed policymaking and consider it vital to effective health services management and planning, they face significant constraints in accessing evidence-specific resources and receive little organizational support. Administrations rely on external capacity to compensate for these limitations and engage with evidence pragmatically.

**Conclusion:**

Our findings indicate moderate and improvable capacity for evidence use in Swiss health administrations that place limited value on organizational support. Besides strengthening organizational support, leadership buy-in, particular staff needs, and balancing the implementation of specific measures with the provision of more general resources should be considered to unlock the potential of strengthened engagement with evidence.

## Introduction

The motivation to understand the role of evidence, i.e., robust information, data, and research, in health policymaking is inherently driven by the desire to enhance its contribution to policy decisions and implementation thereof, ultimately improving health system outcomes. Consequentially, initiatives and interventions that seek to strengthen evidence-informed policymaking (EIPM) have increased in number in recent years. For example, interventions have sought to support policymakers with better evidence access [1–5], build relationships and networks with research-related stakeholders [6–8], provide facilitating infrastructure and processes [7,9], or strengthen evidence-use skills through training [10,11]. Still, the empirical basis of the effectiveness of these interventions is thin, as there are few experimental and large-scale studies [but see 1,12].

A central focus to strengthen EIPM is addressing the abilities, resources, practices, and procedures needed to engage with and use evidence at the level of policy actors, in other words, the capacity of policymakers and their organizations [13–15]. This so-called EIPM capacity constitutes the capability to access, process, and transmit information [16] and can be considered at the individual level (e.g., skills and experience of policymakers), the interpersonal level (e.g., relationships and networks of policymakers), the level of the organization (e.g., systems, culture and norms), and the broader institutional context (e.g., society and politics) [17]. Aspects of capacity include the availability of tools and systems to support evidence use, the prevailing culture regarding how evidence is valued, and skills to find, assess and apply evidence [18].

As relevant health policy actors, public administrations are a key target group for capacity-strengthening interventions. In turn, administrations depend on the availability of research and data for health services management and planning (HSMP) to foster population health and ensure effective, efficient, and sustainable services [19]. Strengthening administrations’ capacity can support engagement with and use of evidence in HSMP [4,12,20,21] and—so it is hypothesized—may help them to make better decisions with scarce resources.

In Switzerland, a democratic federation built around 26 member states (i.e., cantons), there are 26 government health administrations, each with far-reaching health governance and legal, planning, and management authority [22–24], for example, concerning the planning and regulation of hospitals and ambulatory services. Thus, administrations’ are tasked with many health system functions. They would potentially benefit from measures supporting capacity for EIPM. Such measures can range from the introduction of relatively simple tools (e.g., specific training for better engagement with evidence, granting infrastructure for research access, or providing rapid research response mechanisms [25]) to complex, multi-layered interventions [12].

Planning and effectively implementing EIPM-strengthening measures requires understanding the prevailing conditions, existing resources, processes, and necessities for change as perceived by the policymakers concerned [17,21,26,27]. Previous work on EIPM capacity in Swiss health administrations has mainly been confined to assessing how often administrations commission and use evaluation of policy measures [28]. There is a lack of knowledge on available resources and processes that support EIPM beyond aspects that characterize how widely evaluation is institutionalized in administrations [29]. In addition, research in Switzerland has focused on studying particular policies or specific pieces of evidence [28,30–33] but has not attempted to describe administrations’ fundamental needs and views on the role and use of evidence.

This study aimed to explore and characterize Swiss health administrations’ evidence use and capacity. This is done by 1) assessing administrations’ access to and use of tools and systems that support EIPM on the organizational level and 2) investigating individual policymakers’ (i.e., civil servants’) perspectives and needs regarding evidence use and capacity for HSMP.

### Definition of evidence

In this study, we understand *evidence* as “robust information, data, and research” and use these terms synonymously with “evidence” in reporting and discussing the studies’ findings. Based on existing research [34], we applied a more extended and illustrative definition for the capacity assessment:

*Systematically and transparently conducted and reported analyses. These may originate from academic literature, monographs, books, or gray literature, and include internal studies and evaluations. In this sense, robust information, data, and research is not limited to the work of academics from universities but may include findings/studies from other research organizations, e.g., independent research institutes, competence centers, and evaluation and consulting firms*.

## Materials and methods

### Study design

This mixed-method interview study investigated policymakers in German-speaking Swiss cantonal health administrations. Here, policymakers are civil servants (we use the two terms interchangeably) working either as secretary-general or person in charge of HSMP. The sample was limited to German-speaking cantons to ensure that the first researcher to conduct the interviews was able to fully apprehend the opinions and experiences expressed by respondents, both culturally and linguistically.

We quantitatively assessed the organization-level capacity for EIPM by interviewing health administrations’ secretaries-general, applying a measurement tool. To embed these findings in the practical context and investigate individual-level needs and perspectives regarding EIPM, we purposefully selected civil servants responsible for HSMP for in-depth interviews that were subjected to qualitative analysis. Participants were recruited and data collected between October 2020 and May 2021.

### Study setting

The supreme governing body of each health administration, politically and organizationally, is the executive, i.e., one of the five to seven members of the cantonal executive council (the government), a politician elected by citizens. The organization of the administrations differs among cantons. While some executive councils head distinct health departments, others head departments responsible for multiple areas, such as health and social affairs, with a specific unit or section dedicated to health. General secretariats are the staff units and support the executive council in political and operational management of their (health) administration. They are the central interface between politics and administration and are often responsible for areas such as finance and controlling, legal services, human resources, communication, or IT. While every administration has one secretary-general, the number of civil servants concerned with HSMP varies considerably, depending on the canton size, and ranges from a handful to several dozen civil servants. The population of Swiss cantons is between 16,000 and 1.5 million.

### Quantitative capacity assessment

#### Measurement tool

We applied the Organizational Research Access, Culture, and Leadership (ORACLe) [35], a theoretically grounded instrument to assess the existence of supporting systems and tools that facilitate evidence use [12,18,36], hereafter referred to as simply “capacity.” For example, ORACLe assesses the availability of documents that encourage engagement with evidence, training opportunities for evidence access and use, dissemination of evidence, research access resources, and relationships with research. It was developed for interviewing one individual who can provide information representative of an organization or entity. ORACLe consists of a structured 23-question interview and a three-point scoring guide to measure organization-level capacity in seven domains [35]. The development of the domains and the interview questions is based on a literature review, draws on a research-based framework on evidence use [18], and is informed by interviews and iterative interactions with policymakers [37]. We carefully translated the interview and scoring guide from English to German (see **S1 Table**).

#### Participant selection and data collection

Secretaries-general from Swiss health administrations with German as one of the official cantonal languages and a population larger than 50,000 (n = 16) were contacted for interview participation. This arbitrary cutoff was chosen because several of the resources surveyed were likely nonexistent in very small health administrations consisting only of a handful of civil servants.

We chose the secretaries-general as the target persons for the ORACLe interviews because due to their function and position they have a good overview of the administration and its resources. For this reason, secretaries-general also served to identify suitable in-depth interview candidates (see “qualitative in-depth interviews”).

ORACLe questions, domains, and key definitions (i.e., evidence, policy, and policymaking) were provided to all candidates before the interview for preparation purposes. Interviews were conducted and recorded during the COVID-19 pandemic via telephone in Swiss German by the first author. Interviews started with discussing the key definitions. At the end of the interview, we asked for a referral to potential interview candidates for complementary in-depth interviews.

In total, we conducted six ORACLe interviews with secretaries-general from six cantons, with a mean length of 48 minutes. The response rate was 38% (target population: n = 16). Civil servants who were not available for interview participation stated the high workload due to the COVID-19 pandemic as their reason not to participate.

#### Data processing and analysis

Interview recordings were transcribed in the intelligent verbatim fashion for data triangulation with in-depth interviews where the focus is congruent. Overall and domain-specific capacity scores were calculated using the scoring guide (see **S1 Table**) as described elsewhere [35]. For the calculation of the overall capacity score, ORACLe weights each domain differently, according to experts’ opinions on the relative importance of the domain capacity to EIPM. We additionally calculated the unweighted overall capacity scores for comparison of the results.

An additional researcher, trained in rating and otherwise not involved in the project, independently double-scored all interviews to ensure consistency. Disagreements in scoring were resolved through discussion.

We used StataCorp Stata 15 software to calculate domain and overall capacity scores and display them as bar plots. ORACLe data are reported narratively and supplemented with information from the interview transcripts.

### Qualitative in-depth interviews

#### Semi-structured interview guide

We developed a semi-structured interview guide with open-ended questions, probes, and prompts (see **S1 File**) to gain additional insights on evidence use and capacity from an individual perspective and situate the findings in the practical context of HSMP. The interview guide was designed to cover the main themes and concepts concerning the research question and the capacity assessment. It was predominantly developed through a thorough literature review and informed by input from researchers and policymakers. The guide was piloted for comprehensibility using two participants. We aimed to refine the interview guide during data collection based on emerging findings but only made minor changes to the phrasing, with no alterations to the content.

#### Participant selection and data collection

We purposefully sampled civil servants from the higher hierarchical level responsible for HSMP from all German-speaking cantons (n = 21). We have not selected secretaries-general for the in-depth interviews because their scope of work is not limited exclusively to the health sector (e.g., for departments of such as “health and social affairs”) and because their overarching function places them too far away from HSMP practices and decisions.

Interview candidates were identified through health administration websites and ORACLe interviewees’ (i.e., secretaries-general) nominations. A summary of interview topics was provided on request. All interviews started with the provision of key definitions (i.e., evidence, policymaking), followed by the questions of the semi-structured interview guide, and ended with capturing sociodemographic data. 

Face-to-face or video interviews were conducted in Swiss German, audio was recorded in both cases, and notes were taken. Due to the exploratory character, we did not strive for complete thematic saturation [38] but expected to reach this point between ten and fifteen interviews, as the target audience was judged relatively homogeneous [39].

In total, we performed 12 interviews with civil servants from 10 cantons (see **Table 1)** responsible for HSMP, with one additional respondent working in the area of prevention (length range: 40–80 min, mean length: 55 min). Civil servants who were not available for interview participation stated the high workload due to the COVID-19 pandemic as their reason not to participate.

**Table 1:** Qualitative in-depth interview participant characteristics.

Characteristics	*n* or mean (% or range)
**Sex**	
Female	5 (42%)
Male	7 (58%)
**Age**	51 (39−62)
**Highest academic qualification^1^**	
No academic degree	1 (8%)
Master/Bachelor	9 (75%)
PhD	2 (17%)
Continuing education certificate at university	8 (67%)
**Work experience in research**	
No	10 (83%)
Yes	2 (17%)
**Work experience in administration**	
Years in current position	9 (3−28)
Years in administration	14 (3−28)
**Hierarchical level**	
Head of health agency^2^	3 (25%)
Head of health services division	9 (75%)
*of which health agency deputy*	*4 (44%)*
**Number of subordinates**	11 (0−60)

^1^ Multiple answers were possible.

^2^ Highest civil servant responsible for all areas of “health”.

#### Data processing and analysis

All interviews were transcribed verbatim. NVivo 12 was used to facilitate the organization, coding, and analysis of data. We thematically analyzed interview data using the framework method, an approach suitable for policy research that allows the use of qualitative and quantitative data and lends itself to comparative analysis [40,41]. We applied a combination of inductive and deductive analysis to develop the coding framework through open coding while drawing on the existing literature on EIPM and major topics from the interview guide. A preliminary coding framework based on the first four transcripts was developed and then systematically applied to all transcripts by author AB. Concepts not covered by the framework were recorded under new codes and integrated into the final framework once all interviews were coded.

The final framework consisted of 78 codes clustered into nine main categories (i.e., actors and collaboration; needs; attitude and feelings; challenges; potentials and suggestions; strengths and resources; relevancy of evidence in administration; dealing with and using evidence; health service areas). For example, the category “relevancy of evidence in administration” encompassed codes such as “organizational culture,” “focus of the management level,” “self-conception of evidence-informed work” or “pragmatism vs. evidence.” **S2 Table** presents a framework coding example with quotes for the codes of the category “relevancy of evidence in administration”. 

We used the Standards for Reporting Qualitative Research guidelines to report the present study [42]. Data are presented without naming health administrations. To preserve anonymity, we use the letters A−K instead. For participant details, numbers distinguish between civil servants of the same administration. SG stands for secretary-general. All translated quotations were reviewed by a native English speaker fluent in Swiss German. This person was not an author/researcher or otherwise involved in this study.

To minimize potential biases and their impact on the data collection and analysis, we examined our assumptions critically and aimed to gather diverse perspectives. AB is a psychologist and PhD researcher. KW is an epidemiologist knowledgeable and experienced in researching diverse contexts of health management and policy.

### Ethics

This study underwent an ethical review and clearance (Req-2018-00460, response from July 5, 2018) with the Ethics Commission of Northwestern and Central Switzerland. Participants were informed about the study’s purpose and objectives. Written informed consent for study participation and verbal agreement to interview recording were obtained before conducting the interviews. The first author was able to identify individual participants during and after data collection. Interview recordings and transcripts were stored on a password-protected computer and server. The data will be retained for five years after publication of the study results.

## Results

### Moderate evidence-use capacity in health administrations

The overall capacity (scale: 0−9), as assessed by the ORACLe measurement tool, was moderate and similar among the administrations. The mean overall capacity in numbers was 5.1 (range: 4.4−5.8), whereas zero means no and nine means high capacity. For a visual representation, see **S1 Fig.** Calculation of the unweighted total (i.e., the sum of the domain scores, making no assumptions about the relative importance of domains) resulted in a similar pattern, though with a slightly different rank order. Details are presented in **S2 Fig.**

**Fig 1:**
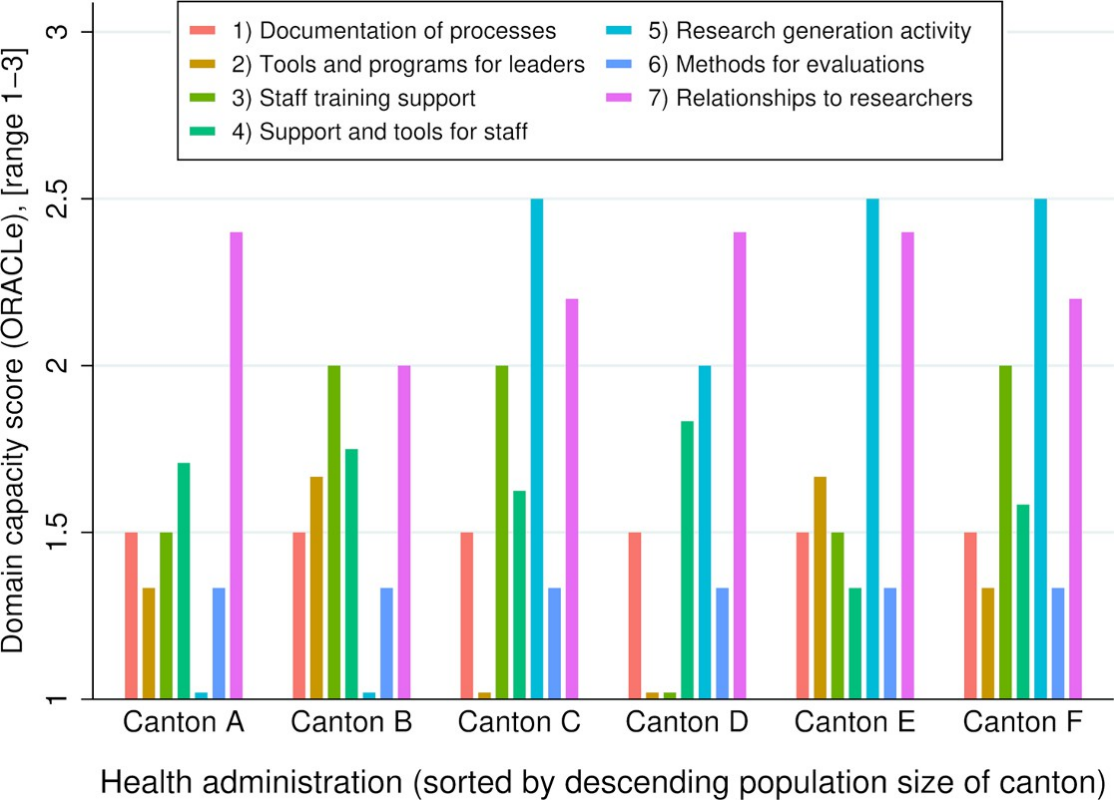
Domain capacity scores by health administration.

Regarding the administrations’ capacity on the level of the seven ORACLe domains, administrations expressed a similar pattern with high and low capacity, generally with considerable variation among domains within an administration (**Fig 1**). Most administrations had the highest capacity scores for their research generation activity (e.g., regarding health services demands and prognoses) or their relationships with researchers and academic partners. Secretaries-general typically reported limited capacity in the documentation of processes, availability of tools and programs for leaders, and staff support with training. As such, administrations scored lowest in domains two to four, which were weighted strongest in calculating the overall capacity score [35]. The following paragraphs inspect the administrations’ domain capacity in more detail.

Administrations indicated uniformly low capacity regarding the documentation of processes that mandate or encourage evidence-use to develop policies (domain one). Documentation was generally limited to formal aspects of politico-administrative processes, such as legislative procedures. There were no examples of written guidance to develop policy or program content. Most explicit requirements were described as being ad hoc on a case-to-case basis. At the same time, a larger scope of the policy or program (i.e., its costs, duration, and extent) increased the relevancy of consulting and demonstrating evidence use, often through the involvement of external partners such as academic institutions or consultancy agencies.

Administrations also exhibited little capacity concerning tools and programs that assist leaders in supporting evidence use (domain two). In most administrations, there were no specific programs for leaders to enhance confidence or expertise in evidence use, and neither job descriptions of collaborators nor performance evaluations/targets entailed expertise in evidence use.

In contrast to the first two domains, capacity in programs enabling staff to learn and maintain evidence-use skills (domain three) varied more strongly among the respondents. Three administrations showed moderate capacity scores, two minimal, and one no capacity at all. Administrations generally provided regular access to training opportunities, and applying evidence to policymaking was sometimes part of the offering. Participation in such training was frequently not considered in staff performance management. Administrations generally reported continuing education and training to be expected but rely mainly on the staff’s initiative.

Administrations showed a similarly low capacity for systems and tools that support staff using evidence (domain four). All but one administration reported regular internal events for the dissemination of evidence. No resources were available that guided access to, appraisal of, or application of evidence. Most administrations indicated having staff expertise in evidence use. While the expertise was required or assumed for certain functions, it was not tied to a specific role. In general, this expertise was described as being accessible to other employees. Secretaries-general also stated that administrations had access to most or all relevant academic journals. They also indicated that needs and, thus, access differed significantly among individuals and different fields of work. On the other hand, databases with research literature were available only in some administrations. About half the administrations had an easily accessible and cataloged library, and none systematically provided licenses for literature-management software. There were no established methods to commission literature reviews or knowledge-management systems specifically applied for evidence management.

Secretaries-general indicated the highest capacity scores for efforts to generate new evidence (domain five). While four of six administrations showed moderate to high capacity, two indicated none. Most research projects were carried out with the involvement of external partners. Half the administrations reported recently having commissioned one or more research projects.

Capacity regarding processes for evidence-informed policy evaluations (domain six) was uniformly low among all administrations. Although evaluations for measures of certain scope were expected and carried out regularly or frequently, a requirement for undertaking such evaluations was not explicitly documented.

All administrations showed moderate to high capacity regarding their relationships with researchers and research organizations (domain seven). Administration staff generally participated in research fora or conferences, and most administrations had several formal and informal relationships with academic organizations. There were no administration-staff appointments from research organizations in the area of health services. However, there was regular consultation and involvement of external experts in administrations’ work, often service providers, such as physicians, with double affiliations to practice and academia.

### Potential for increased organization-level, structural capacity in health administrations

When asked about the evidence base for HSMP, civil servants frequently criticized data and information gaps in some areas of work along with challenges in accessing data (“we really have to scrape it together”—J1; “there we are groping around completely in the dark”—A1). While the availability of inpatient care data is typically considered satisfactory, the “black box” of outpatient data presents a challenge due to poor comprehensiveness and quality (F1), making it intricate to assess important trends, such as the shift of inpatient care services to the outpatient sector. Similarly, outcomes data related to the quality of care would be necessary for civil servants’ work in governing health care but hardly exist. Individual voices criticized “statistical silos” and emphasized the potential value of better linking data collected through different systems—for example, health and social care data (D1).

There is not only a lack of data but also of personnel, expertise, and time to analyze available evidence. Due to limited resources, civil servants felt they did not have enough time to deal with specific problems in greater depth or study the academic literature.

*Lean management means efficient reduction [of activities] to the core business [*…*] not prospective planning and projects … that is a bonus*. *So*, *the more you are under pressure with resources*, *the more you have to reduce [the time you devote] to the day-to-day business*. *And I think that’s not just in our office*, *but that’s the situation in the administration in general*. D1

In this context of scarce resources, civil servants are forced to use evidence pragmatically. Data and information are sought and processed when they are available and “accessible at a reasonable cost,” and their contribution is judged to be meaningful (H1). Consequentially, HSMP decisions rely on minimal information and intuition. Where the administration can manage a task effectively, there is little incentive from an organization-level perspective to examine the validity of underlying data more closely.

Apart from the lack of general resources, the pragmatic engagement with evidence is a consequence of the low institutionalized EIPM-specific support and guidance in the administration. Despite leadership typically supporting EIPM, administrations place the focus and responsibility for engagement with evidence at the level of individual civil servants that “should be able to work as freely as possible” (H1). Thus, administrations show limitations in structural capacity.

While the administrations’ prevailing informal culture towards EIPM is considered relevant for individual civil servants’ evidence-use behavior, this behavior is perceived as being influenceable only to a limited extent, as one secretary-general outlines:

*People just have different ways of doing things*. *Some find it exciting and like to read such documents; others do it less [gladly]*. *You can’t enforce this very well*. *You can’t tell someone that they have to read three academic publications on a certain topic every month*. *You can’t do that at this level*. *It’s more about having that culture and also keeping the curiosity to know what’s happening in the specialty and what exciting things have come out of the research*. A-SG

When asked about their needs for the administration’s support in evidence management, the civil servants provided few specifics, indicating that there is no conception of potential targeted EIPM support measures. Apart from the desire for research databases access in two administrations, civil servants said that they require resources such as time and additional staff, more orientation to and overviews of existing data, health service statistics, academic publications, and health system guidelines.

The administrations depend on external resources to compensate for their limited internal capacity to produce and engage with evidence. An essential such resource for health care planning, especially for medium-sized and smaller cantons, is the government-supported competence center Swiss Health Observatory (OBSAN; https://www.obsan.admin.ch/en). The OBSAN offers analytical expertise, provides access to otherwise difficult-to-access data, and produces valuable evidence for policymaking. It serves, to some extent, as a knowledge broker (D1) that “can reconcile the balancing act between” politico-administrative needs and scientific demands (H2) and helps administrations that are “overwhelmed” (H1) with data use in dealing with their limited internal capacity.

As also noted in the capacity assessment, the in-depth interviews confirmed collaboration with research organizations and research-related consulting firms to be relevant to the administration. Nevertheless, such collaboration is sporadic, with little direct contact overall. While some civil servants emphasized that research and evaluation assignments are “horribly expensive” (F1), others regretted that “you just can’t spend money if you don’t have time” (A1) to manage an evaluation mandate.

Besides that, health care providers—first and foremost, hospitals—are vital to the administration in building a “bridge between practice, research, and health authorities,” providing access to evidence and supporting the administration in its appraisal (A-GS). These “experts who are on the front lines” (G1) are essential partners in aligning, planning, and implementing health care policies. This relationship constitutes a knowledge asymmetry in favor of the service providers and is thus not without problems. Especially since service providers do not primarily think in terms of a networked and integrated “health care logic for the population” (A1). An interviewee from a large canton expressed the hypothesis that in the administration’s efforts to manage the service providers better, the need for evidence in the administration has increased (G1).

### Evidence is strongly valued for health service management and planning

Interviews have demonstrated that evidence gives civil servants confidence in and orientation for actions, helps them understand the current healthcare situation, identifies areas in which action is needed, allows forecasting of future needs, and drives planning. Overall, evidence is becoming increasingly crucial to the administration’s work to shape health care. External demands, such as those from the government, parliament, and citizens, require the administration to employ evidence, as outlined by the civil servants. Some institutionalized political processes, such as reporting to parliament or legal requirements, even oblige using evidence.

*I think evidence is very important for us in our daily work but also in health care planning*. *If we have to assess the health care situation*, *we need information*, *data*, *evidence*, *we need to know if access is guaranteed—if they [the patients] can see a general practitioner within such and such a time*, *for example*, *or a hospital*, *and so on*. K1

Besides the relevance of evidence for effective HSMP from an organization-level perspective, civil servants consider the employment of evidence important and situations with little or unclear evidence unsatisfactory. They wish existing evidence to be consulted more frequently within decision processes and desire opportunities to perform more in-depth analyses of topical areas they are tasked with. Civil servants’ narrative suggests that efforts to incorporate evidence into the work “as well as possible” (F1) is a consequence of their self-image in the sense of a “professional self-expectation” (D1) and “attitude” (A1). Thus, accounts of using evidence as working “well and carefully” (A1) highlight the internalization of evidence’s fundamental importance and its normative meaning as the right guide to make decisions about HSMP.

The area of hospital planning is perceived to be particularly evidence-informed, and for care planning in general, the medical statistics of the hospitals to represent the “basis of the whole” (E1). Population, hospital, cost, social security data, and other forms of evidence, such as academic publications and survey data, also feed into policy- and decision-making. However, the academic literature appears less relevant for HSMP—it is used to answer specific questions, such as how health care for medical conditions like strokes should be provided or how minimum case numbers should be used to steer care. Regarding the application to health care governance in Switzerland, the international research literature is often considered “not useful for our conditions or our problem” (H2).

*Only studying the literature doesn’t help that much either*, *so if you read any [studies] from Germany or England* … *we don’t have an NHS (National Health Service)*, *and we don’t have the same underlying circumstances in terms of funding as Germany or so*. *Thus*, *certain things you just can’t realize*. A1

The same argument is made about the transferability of evidence or policy solutions between cantons, which is considered limited due to contextual situations and environmental differences, such as geography.

Other civil servants emphasize the potential of intensified evidence and tacit knowledge exchanges between the cantons. In health care planning, such interactions are described as close in isolated cases but limited overall. Essentially, they depend on individuals in the administration, predominantly involve the nearest neighboring cantons, and hardly exist across language borders in Switzerland.

From a theoretical point of view, the conceptual use of evidence [43]—for example, to develop new ideas for health services and their regulation—seems to be less predominant than, for instance, symbolic uses to legitimize preexisting positions [44]. Indeed, civil servants highlighted that evidence supports their arguments within the administration and discussions with external policy actors, such as service providers or professional associations, supports the justification actions, and helps convince stakeholders or enforce plans.

### High individual-level motivation to engage with evidence despite a challenging politicized context

The administration work occurs in a “political environment” (B1). Therefore, administrations can only shape EIPM to some extent, for example, by providing information and creating framework conditions to promote the integration of evidence through their role in managing service providers. The role of the administration was described as “to do a balancing act” between “those who are concerned, science and politics” (H2) and trying to “promote evidence-based policy” (J1). As individuals who help the administration carry out this role, they see it as their duty to alert when policy proposals conflict with evidence. In some cases, this conception of the role goes so far that civil servants bring evidence into the political processes beyond the administration’s management level if it is in danger of being withheld there. Here is how one civil servant describes such actions:

*By working on it and making the evidence available to different political stakeholders … and with that*, *the possibility was actually no longer there to just let the [evidence] disappear into the drawer*. *That was not always without its problems*, *that conduct*. H1

Civil servants understood and accepted political rationales but sometimes described being dissatisfied with the limited inclusion of evidence in political processes. They identify patterns that resembled a dichotomy [45] between substantially evidence-informed work on the part of the administration, at least up to the political-strategic level, versus the political decision-making arena, in which evidence does not play an essential role and policymaking is often driven by ideology and expected short-term benefits.

At the same time, respondents provided several examples where evidence significantly influenced or shaped policymaking. Still, their discussion of health policy was dominated by a narrative on the limited impact of evidence on the political rationales. Local and regional policy context was generally described as more ideology-based and conflicting with EIPM.

*The more local [the policy issue] the less*, *how should I say … evidence-based*, *data-based it is*, *because those data are not available in studies or anything the like*. D1

In this politicized context, using evidence to advocate for a cause can result in negative consequences. A few civil servants reported being verbally attacked professionally or even personally for ideological reasons in advocating for evidence, given “a roasting” (A1) or “finished off” (H2).

The discrepancy between political motives and evidence becomes particularly apparent in the case of recommendations to discontinue ineffective programs, such as disease screening, or cuts to oversupplied services and infrastructure, such as hospital closures.

*You could say that health care*, *accessibility*, *remains just as good*, *but the quality could increase*, *and the costs are better controlled*. *These are not always the arguments that work when it comes to a local vote on whether a population wants a hospital close to home or not*. *There are completely different emotional and*, *economic*, *local aspects involved*, *which then prevent this*. G1

## Discussion

Healthcare governance is becoming increasingly complex and requires more than ever the incorporation of information, data, and research to find effective and broadly supported solutions to health systems’ challenges. This study, relying on interviews with civil servants, explored evidence use, capacity, and related needs and perspectives in Swiss health administrations in HSMP. The findings contribute to a better understanding of the prevailing context for introducing EIPM support measures in health administrations.

Interviews revealed that administrations would particularly benefit from implementing EIPM-specific structural measures at the organizational level. For example, introducing programs supporting evidence use or aligning administration processes to that aim could help dealing with currently limited support and guidance for EIPM. Administrations seem to place the focus and responsibility regarding the engagement with evidence on individuals and offer little specific support for EIPM. The findings of this study also suggest that allocating more “general” resources, such as work time, could drive engagement with evidence for HSMP—a finding to be taken into account for EIPM-strengthening considerations also for person-based and expertise-focused policy advisory systems in countries such as Germany and Italy [46].

Our results show that civil servants are committed to EIPM, value evidence for their work, and can be considered the foundation and substrate for EIPM in health administrations. Thus, providing adequate resources is a prerequisite for meeting civil servants’ needs and motives regarding EIPM in HSMP. As general resources build the basis for engagement with evidence in the first place and determine the potential of EIPM-specific support, reflections on implementing EIPM support should consider strengthening general resources alongside targeted measures. For example, we found that civil servants demand more and better quality healthcare data for effective system governance. However, making use of such data requires time and knowledge. Without basal resources to understand and analyze these data, isolated investments in enhanced data availability and access will be of little value [47,48]. Further research should show how the focus on (general and EIPM-specific) resources and support varies between the individual and organizational levels in other countries, especially in European and federally organized countries.

Our study showed that administrations are compensating for the lack of internal resources and competencies for EIPM by drawing on external capacity, for example, by commissioned analyses or reports with research and consulting offices—a consequence of the vital role of private actors in Swiss health policymaking and the relatively lean staffing in administrations [23,49,50]. The support of the OBSAN best demonstrates this in analyses and health care planning, which is both required and highly appreciated by many cantons [51,52]. Outsourcing capacity may be instrumental where fast results are needed, projects are large or highly complex, or administrations lack skilled personnel. Building internal capacity instead of relying on external services may promote EIPM beyond addressing concrete and immediate practical issues, for example, by fostering conceptual evidence use through a research-affine environment [43,53]. Internal capacity-building may also help the administrations critically review and interpret the evidence provided by other health system actors, support the assessment of policy measures, and ensure their efficient implementation [5,15,54]. Beyond that, administration leadership and civil servants currently possess little knowledge of potential EIPM-supportive measures. Building internal capacity and organizational processes might help them make better use of existing tools and services [4,5,55,56].

This study aimed to assess the EIPM capacity of health administrations. We identified moderate evidence-use capacity in Swiss health administrations that exhibit a similar profile of domains with strong and weak capacity. Surprisingly, regardless of their size, the administrations’ overall capacity was comparable in magnitude. In light of the existing literature on the relationship between administration size and the use and institutionalization of evaluations [30,31,57,58]—as a specific form of evidence and thus an indicator of EIPM—these results are somewhat surprising and require further clarification.

Most secretaries-general indicated that the administration had close formal and informal relationships with researchers. The in-depth interviews qualified this finding. Nevertheless, contact was characterized as limited and sporadic because it specifically happens within larger joint projects, which are rarely carried out due to the number of resources they tie up. A more detailed assessment of the administration-research relationship with ORACLe could provide a more precise picture of strengths, weaknesses, and potentials in this regard. Understanding this relationship is particularly relevant since study findings indicate that existing resources could be used more efficiently by intensified cooperation between administrations to initiate larger-scale, cross-cantonal studies or commission jointly funded contract research.

This study confirms that policymaking about HSMP happens in a politicized environment with many different actors, interests, and values [32,59–64]. Concerning the implementation of EIPM support measures in health administrations, this finding suggests that fostering the engagement with and use of evidence depends on the buy-in of administration leaders [5,65,66], be it straight-forward measures such as promoting or demanding the use of evidence in administrations processes and mission statements, to more complex changes like adapting the organizational culture towards EIPM [4,5,20]. Thus, future research will have to show how administration staff can influence their political leaders in a way that investing in EIPM serves the needs of individual civil servants and the administration’s agency, effectiveness, and impact [67].

An alternative way of supporting EIPM that depends less on the endorsement of administration leaders is the investment in the already established relationships and services with organizations that currently provide capacity [68,69]. Given the credibility and usefulness of evidence generated by the OBSAN, one could consider expanding its role and providing it with more financial resources and tasks. Ideally, such investments are coupled with efforts to institutionalize part of the externally provided capacity [70].

The COVID-19 pandemic has shown that structured partnerships between government and research are essential for rapid knowledge exchange and the development of evidence-informed policies [71–74]. Defined or institutionalized forums such as advisory panels may help administrations better consult evidence and involve experts more directly in policymaking [46,75]. In the context of these partnerships, the growing availability of structured support tools for evidence-based decision-making on complex health system decisions is also of interest. While such structured decision-making processes may not be suitable for all policymaking activities, there are areas of public health policy and practice where they offer an opportunity to make more effective use of evidence [76,77].

The findings of this study highlight how essential evidence is to the daily work in planning and securing health services by administrations. We found that civil servants particularly require health service data and statistics [78], and the promotion and accessibility of health data are essential for further developing HSMP [79,80]. On the other hand, research evidence was confirmed to have primarily limited relevance in daily work [78]—a finding relevant for developing and implementing future measures to promote EIPM. Not surprisingly, one explanation for the low value placed on academic literature may be the difficulty of applying foreign studies to the local context [81], as stated by several interviewees. Since administration staff struggles with applying research to real-world problems, research organizations and federal agencies could further drive EIPM by contextualizing international data and studies, identifying possible policy measures for adoption, and outlining implementation considerations in local settings [4,68,82,83]. Thus, reflections on strengthening EIPM would benefit from a holistic perspective highlighting system needs for evidence use capacity and requiring multiple stakeholders’ involvement.

### Limitations

This study targeted a specific, comparatively small group of policymakers from German-speaking state-level health administrations and focused mainly on HSMP at the intermediate to high managerial level. The specificity of the sample might limit the transferability of findings to other work areas within health administrations and language regions in Switzerland and beyond. For example, French-speaking Swiss cantons lean toward a stronger role of the public sector, with the governmental services at the forefront in areas such as public health [84,85]. Such cultural differences among the language regions might manifest in the extent administrations support EIPM and provide respective resources [but see 30]. However, as participants were from administrations representing diverse cantonal characteristics, we consider central issues for Swiss health administrations to be captured. In addition, the descriptions provided by the interview participants correspond broadly with findings from the international literature, suggesting that the results of this study are also relevant to other countries and contexts.

It must be noted that the capacity data presented is based on information provided by six health administrations, with each participant representing one administration. It is possible that the secretaries-general interviewed might not have been aware of all details concerning evidence in specific administration areas [86]. Moreover, due to the exclusion of very small cantons with less than 50,000 inhabitants (n = 5) from the capacity assessment, an underestimation of the mean overall capacity cannot be excluded. Therefore, the general validity of the results should be interpreted with caution. Future studies should verify and extend these results with a broader target group and a larger sample, preferably with quantitative surveys in written or electronic form [e.g., 87].

Several capacity-measurement tools are available [e.g., 88–90]. We selected ORACLe because its development was strongly guided by academic literature and extensively informed by policymakers and knowledge-translation experts. The tool provides clear operationalization of capacity magnitude and tool availability, targeted toward health-policy organizations. Moreover, ORACLe was developed for and tested in a high-income context and found helpful [12,37]. However, the application of the capacity-measurement tool showed the potential for improvement, as recently confirmed by other scholars [91]. For example, we found that interview questions might benefit from a more detailed operationalization of the concepts surveyed. Furthermore, the specificity of the scoring guides’ categories could be enhanced, as assigning interviewee responses to the categories proved challenging in some instances. While these issues should be addressed in future applications of ORACLe, we mitigated shortcomings in the measurement and improved consistency by consulting a second independent rater who double-coded all interviews.

Finally, due to the heavy workload of health administrations in responding to the COVID-19 pandemic, such a quantitative approach involving a large group of civil servants was not considered ethical and operationally feasible. Indeed, this study was conducted in a pandemic context that strongly influenced the operations of cantonal health administrations. As data collected were self-reported, it cannot be ruled out that the salience of research in the pandemic context has impacted current perspectives on the relevance of evidence and administrations’ resources to engage with it. Similarly, social desirability bias cannot be ruled out but seems unlikely, as interview participants felt generally comfortable expressing criticism of their administration, shortcomings in their performance, or its resources.

## Conclusion

This study found moderate capacity for EIPM on HSMP in Swiss health administrations. Findings indicate potential for capacity-strengthening measures and existing opportunities for implementing EIPM support. The individual civil servants of the administration are committed to EIPM and value evidence for HSMP, whereby they prefer health service data and statistics over research evidence. But scarce resources for EIPM and limited organizational support and guidance constrain enhanced engagement with evidence in daily practices. Presently, the focus and responsibility for EIPM in HSMP remain with individual civil servants. These demonstrate the need for EIPM support to carry out effective work that depends on the external capacity to compensate for an internal lack of resources. To unlock this potential in health administrations, the assessment of EIPM support options should consider building capacity on the organization level and pay attention to leadership buy-in and specific staff needs. Considerations about capacity-strengthening may benefit from balancing the implementation of EIPM-specific measures with the provision of more general resources.

## Supporting information

S1 Fig Total capacity scores by health administration. Green bars display the overall capacity score per investigated health administration, as assessed with ORACLe.
(TIF)

S2 Fig Unweighted total capacity scores by health administration. Blue bars display the unadjusted overall capacity score per health administration, as assessed with ORACLe. Unadjusted means that domains of ORACLe are not assigned weights and are therefore considered equally relevant for calculating the overall capacity.
(TIF)

S1 Table ORACLe German translation. (DOCX)

S2 Table Framework coding example. Exemplary quotes for codes of the category “relevancy of evidence in administration” of the final framework.
(DOCX)

S1 File In-depth interview guide. (DOCX)

## Abbreviations

COVID-19: Disease caused by the novel coronavirus SARS-CoV2
EIPM: Evidence-Informed PolicyMaking
HSMP: Health Services Management and Planning
OBSAN: Swiss Health Observatory
ORACLe: Organizational Research Access, Culture, and Leadership
