# Supplementary figures and images for: Exploring evidence use and capacity for health services management and planning in Swiss health administrations: A mixed-method interview study
Source: PLoS One. 2024 May 8;19(5):e0302864. doi: 10.1371/journal.pone.0302864 (PMC11078391; doi:10.1371/journal.pone.0302864)

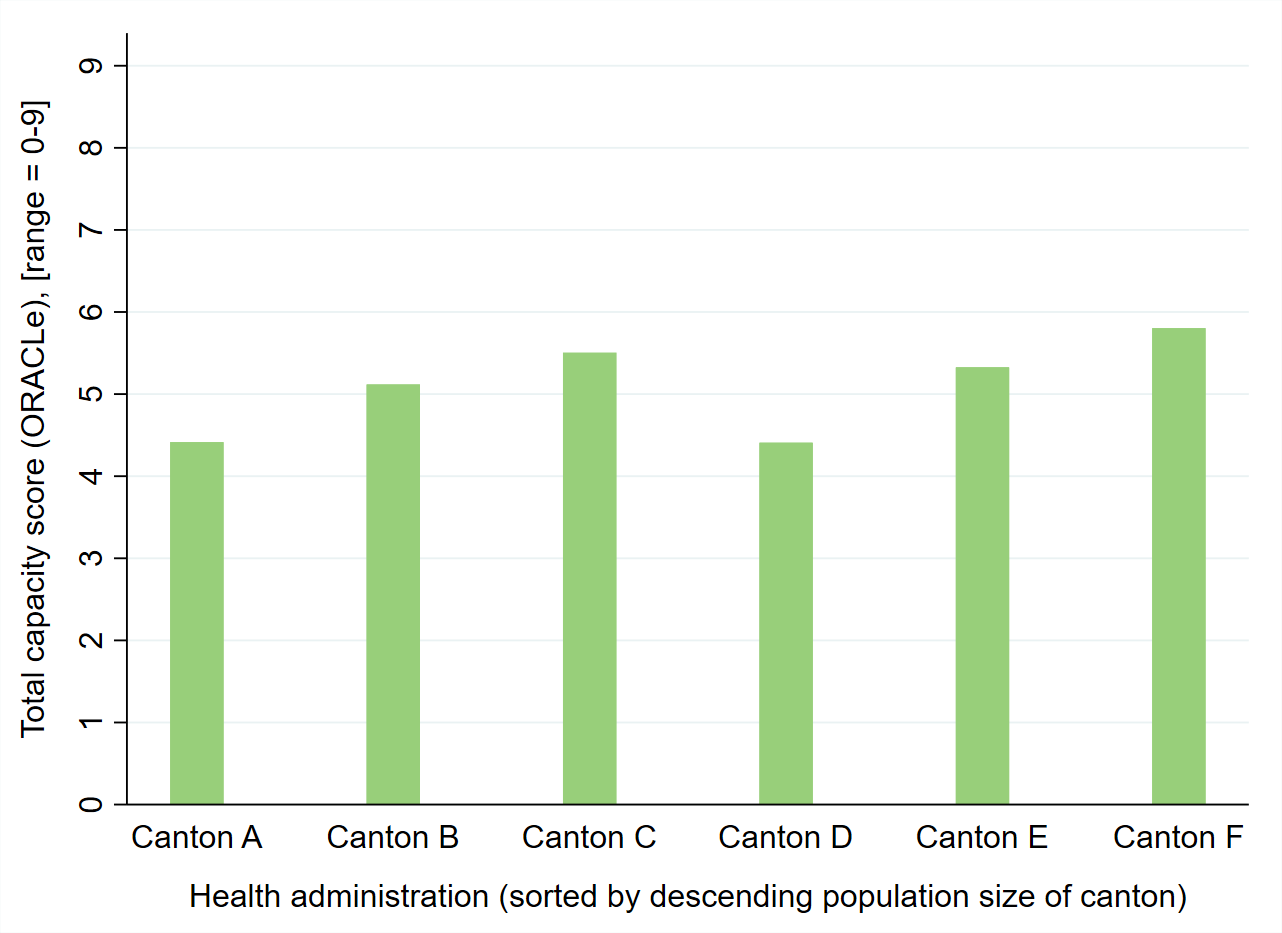

Supplement: S1 Fig — Green bars display the overall capacity score per investigated health administration, as assessed with ORACLe. (TIF) [file pone.0302864.s001.tif]
