# Supplementary material for: Exploring evidence use and capacity for health services management and planning in Swiss health administrations: A mixed-method interview study
Source: PLoS One. 2024 May 8;19(5):e0302864. doi: 10.1371/journal.pone.0302864 (PMC11078391; doi:10.1371/journal.pone.0302864)
Supplement: S1 Table — (DOCX) [file pone.0302864.s003.docx]

## S1 Table. ORACLe German translation.

| Interview-Frage | SPIRIT Bereich | Bewertungsschema | | |
| --- | --- | --- | --- | --- |
|  |  | ja, sogar sehr (3 Punkte) | teilweise/begrenzt (2 Punkte) | nein (1 Punkt) |
| 1. Verfügt Ihre Verwaltung über dokumentierte Prozesse, wie politische Massnahmen oder Programme entwickelt werden sollten?  🡪 Falls *keine* weiter zu Frage 3 | Bereich 1: Dokumentierte Prozesse, die den Einsatz von Forschungsergebnissen für die Entwicklung von politischen Massnahmen und Programmen fördern oder vorschreiben | Es gibt standardisierte, schriftliche Anleitungen, die beschreiben, wie politische Massnahmen und Programme entwickelt werden sollten, und diese sind organisationsspezifisch. | Es gibt dokumentierte Prozesse für einige Aspekte der Massnahmen- und Programmentwicklung, aber nicht für alle, nicht auf einem sehr hohen Niveau und mit wenig Details. | Es gibt keine dokumentierten Prozesse. |
| 2. Ermutigen oder erfordern diese Prozesse, dass Forschungsergebnisse in der Entwicklung von politischen Massnahmen und Programmen genutzt werden? | Bereich 1: Dokumentierte Prozesse, die den Einsatz von Forschungsergebnissen für die Entwicklung von politischen Massnahmen und Programmen fördern oder vorschreiben | Die Forderung nach der Nutzung von Forschungsergebnissen muss in der Dokumentation der Verwaltung explizit und unmissverständlich vermerkt sein (entweder als Forderung oder als Ermutigung) und muss die Art und Weise der Nutzung von Forschungsergebnissen sowie die Forderung, dass diese genutzt werden sollten, enthalten. | Die Nutzung von Forschungsergebnissen ist impliziert, in der massgeblichen Dokumentation aber nicht ausdrücklich empfohlen oder verlangt. Oder die Dokumentation beinhaltet nicht, auf welche Art und Weise die Forschungsergebnisse genutzt werden soll.  Impliziert ist es, wenn die Dokumentation auf die Unterstützung von «Evidenz» im Allgemeinen hinweist und nicht auf Forschungsergebnisse im Spezifischen. | N/A wenn Prozesse nicht vorhanden sind.  Nein, wenn es dokumentierte Prozesse gibt, diese sich aber nicht auf die Nutzung von Forschungsergebnissen beziehen. Oder wenn es eine "Kultur" oder die Annahme einer Forschungsnutzung gibt, diese aber in der massgeblichen Dokumentation nicht vorgeschrieben oder gefördert wird. |
| 3. Gibt es Programme für Führungskräfte, um deren Selbstvertrauen oder Expertise hinsichtlich der Nutzung von Forschungsergebnissen in der Politikgestaltung zu verbessern?  Als Führungskräfte gelten Mitglieder der Exekutive oder des Managements sowie andere Personen mit einer formellen oder informellen Führungsrolle. | Bereich 2: Werkzeuge und Systeme zur Unterstützung von Führungskräften, um den Einsatz von Forschungsergebnissen in der Entwicklung von politischen Massnahmen und Programmen aktiv zu fördern | Muss speziell auf Führungskräfte abzielen (und nicht auf Programme, die sich an alle Mitarbeitende, einschliesslich der Führungskräfte, richten).  Diese Programme sollten regelmässig, d.h. mindestens einmal im Jahr, angeboten werden. | Programme für alle Mitarbeitende, einschliesslich Führungskräfte.  ODER bietet einmalige oder gelegentliche Programme nur für Führungskräfte an. | Es gibt keine Programme, an denen Führungskräfte teilnehmen oder die speziell für Führungskräfte gedacht sind. |
| 4. Beinhalten Stellenbeschreibungen oder Leistungsbeurteilungen/Zielvorgaben  von Entscheidungsträger/innen die Expertise in der Nutzung von Forschungsergebnissen bei der Politikgestaltung | Bereich 2: Werkzeuge und Systeme zur Unterstützung von Führungskräften, um den Einsatz von Forschungsergebnissen in der Entwicklung von politischen Massnahmen und Programmen aktiv zu fördern | Die Expertise in der Nutzung von Forschungsergebnissen muss explizit sein und in den meisten Stellenbeschreibungen oder Leistungsbeurteilungen der Entscheidungsträger/innen enthalten sein. (Entscheidungsträger/innen und nicht generell leitende Mitarbeiter) | Die Stellenbeschreibungen oder Leistungsbeurteilungen von Entscheidungsträger/innen können sich auch auf Expertise beziehen, die den Einsatz von Forschungsergebnissen implizieren, aber nicht explizit machen.  ODER die Expertise in der Nutzung von Forschungsergebnissen wird zwar explizit erwähnt, aber ist nur in einigen wenigen Stellenbeschreibungen der Entscheidungsträger/innen vorhanden. | Es gibt keinen Hinweis auf die Verwendung von Forschungsergebnisse in den Stellenbeschreibungen oder Leistungsbeurteilungen der Entscheidungsträger/innen. |
| 5. Haben Führungskräfte Ihrer Verwaltung in den letzten sechs Monaten in ihrer internen Kommunikation auf Forschungsergebnisse hingewiesen (z.B. Newsletter, Bulletins, Tweets usw.)? | Bereich 2: Werkzeuge und Systeme zur Unterstützung von Führungskräften, um den Einsatz von Forschungsergebnissen in der Entwicklung von politischen Massnahmen und Programmen aktiv zu fördern | Dies sollte innerhalb der letzten sechs Monate mindestens einmal pro Monat geschehen sein. | In der internen Kommunikation wird nur unregelmässig und selten auf Forschungsergebnisse verwiesen. Dies erfolgt weniger als einmal pro Monat.  ODER Newsletter oder Mitteilungen gibt es zumindest monatlich, diese beziehen sich aber nur unregelmässig (weniger als einmal pro Monat) auf Forschungsergebnisse | Es gibt keine relevanten internen Mitteilungen oder, falls es doch solche gibt, beziehen sich die Führungskräfte darin entweder nicht auf Forschungsergebnisse oder haben dies in den letzten 6 Monaten nicht getan. |
| 6. Bietet Ihre Verwaltung Zugang zu Fortbildungsmassnahmen hinsichtlich dem Zugang, der Beurteilung und Anwendung von Forschungsergebnissen für die Politikgestaltung? | Bereich 3: Verfügbarkeit von Programmen zur Ausbildung und Aufrechterhaltung von Fähigkeiten für die Nutzung von Forschungsergebnisse in der Massnahmen- und Programmgestaltung | Die Ausbildung sollte spezifisch auf Forschungskompetenzen ausgerichtet sein und nicht bloss im Verlauf anderer Ausbildungen erwähnt werden. Die Verwaltung muss die Ausbildung intern durchführen oder den Mitarbeitenden die Teilnahme an externen Schulungen ermöglichen. Der Zugang zu den Programmen wird den meisten Mitarbeitenden aktiv angeboten, nicht nur auf Anfrage.  Sollten regelmässig, d.h. mindestens einmal im Jahr stattfinden, und für alle zugänglich sein. | Ja, Schulungen werden auf Nachfrage hin angeboten, aber sie werden nicht allgemein angeboten oder unterstützt, und auch nicht auf kontinuierlicher Basis.  ODER  Ja, aber das Personal ist sich dessen vielleicht nicht bewusst.  Es gibt Schulungen zum Zugang, zur Bewertung und/oder Anwendung von Forschungsergebnissen im Allgemeinen, jedoch nicht speziell für die Massnahmen- und Programmgestaltung | Es werden keine internen Schulungen angeboten, und es gibt keine Unterstützung für Mitarbeitende extern an Kursen teilnehmen. |
| 7. Wird die Teilnahme an Schulungen über den Zugang, die Beurteilung und Anwendung von Forschungsergebnissen in der Politikgestaltung in den Leistungsbeurteilungen/Zielvorgaben von Mitarbeitenden berücksichtigt? | Bereich 3: Verfügbarkeit von Programmen zur Ausbildung und Aufrechterhaltung von Fähigkeiten für die Nutzung von Forschungsergebnisse in der Massnahmen- und Programmgestaltung | Die Leistungsbeurteilungen müssen für die meisten relevanten Mitarbeitenden ausdrücklich die Schulung zur Nutzung von Forschungsergebnissen oder Evaluationen erwähnen. | Die Leistungsbeurteilungen decken nur einen oder zwei dieser Aspekte ab, z.B. ist die Anwendung von Forschungsergebnissen oder Evaluation nicht enthalten.  ODER dies wird nur für das Leistungsmanagement einer sehr kleinen Gruppe von Mitarbeitenden als relevant erachtet, z.B. von Mitarbeitenden, deren gesamte Arbeit im Bereich Evaluation liegt, die aber keine regulären Entscheidungsträger/innen sind.  ODER es ist im Leistungsmanagement impliziert, aber nicht explizit erwähnt.  ODER es wird nur dann als Problem betrachtet, wenn sich das verfügen über Forschungskompetenzen als relevant herausstellt. | Die Teilnahme an Schulungen wird beim Leistungsmanagement nicht berücksichtigt. |
| 8. Wurden in den letzten sechs Monaten relevante Forschungsergebnisse (Publikationen, Berichte, Synthesen oder Bulletins) in Ihrer Verwaltung verbreitet? | Bereich 4: Verfügbarkeit von Hilfsmitteln und Instrumenten, die den Zugang zu Forschungsergebnissen und deren Anwendung erleichtern | Dies sollte häufig geschehen, d.h. mindestens mehrmals im Monat, und muss in den letzten sechs Monaten geschehen sein.  Es spielt keine Rolle, wer diese versendet, d.h. durch Kollegen auf einer Ad-hoc-Basis oder mittels einem systematischeren Ansatz. | Dies geschieht weniger als zweimal im Monat. | Relevante Forschungsergebnisse wurde in den letzten sechs Monaten nicht verbreitet oder wurde generell nicht verbreitet. |
| 9. Verfügt Ihre Verwaltung über Ressourcen, die für den Zugang, die Bewertung und Anwendung von Forschungsergebnissen anleiten? | Bereich 4: Verfügbarkeit von Hilfsmitteln und Instrumenten, die den Zugang zu Forschungsergebnissen und deren Anwendung erleichtern | Die Verwaltung muss über dokumentierte Ressourcen (Handbücher, Leitfäden, Online-Lernmodule usw.) zu allen drei Aspekten verfügen und den Mitarbeitenden leicht zugänglich sein. | Die Ressourcen sind begrenzt oder decken nicht alle drei Aspekte der Forschungsnutzung ab. | Es gibt keine dokumentierten Ressourcen. |
| 10. Verfügt Ihre Verwaltung über Mitarbeitende mit anerkannter Expertise hinsichtlich dem Zugang, der Beurteilung und der Anwendung von Forschungsergebnissen für die Politikgestaltung? | Bereich 4: Verfügbarkeit von Hilfsmitteln und Instrumenten, die den Zugang zu Forschungsergebnissen und deren Anwendung erleichtern | Die Expertise muss für die meisten Mitarbeitenden zugänglich sein, ein hohes Niveau haben und an eine bestimmte Rolle gebunden sein und nicht an eine Person, die zufälligerweise über diese Fähigkeiten verfügt. | Die Expertise ist nicht an eine Rolle gebunden. Einige Personen mögen über diese Fähigkeiten verfügen, aber dies ist zufällig und/oder andere Mitarbeitende sind im Allgemeinen nicht in der Lage, auf diese Expertise zuzugreifen. | Nein, niemand solches ist verfügbar. |
| 11. Verfügt Ihre Verwaltung über folgende Ressourcen  i. Abonnemente für Forschungszeitschriften?  ii. Abonnemente für Datenbanken mit Forschungspublikationen?  iii. Eine Bibliothek oder eine elektronische Bibliothek?  iv. Lizenzen für Literaturverwaltungssoftware (z.B. Endnote)  Hinweis: Diese Fragen beziehen sich auf Ressourcen, die von der Verwaltung zur Verfügung gestellt werden, und beinhalten NICHT Ressourcen, die einzelnen Mitarbeitern gehören (z.B. Universitäts-Logins, eigene Lizenzen für EndNote). Wenn dies der Fall sein sollte, wird der Wert 1 vergeben.  Bei dieser Frage werden Punkte für jeden der vier Bereiche (i-iv) vergeben. | Bereich 4: Verfügbarkeit von Hilfsmitteln und Instrumenten, die den Zugang zu Forschungsergebnissen und deren Anwendung erleichtern | i. Themenspezifische Fachzeitschriften - ja, der Zugang zu allen oder den meisten relevanten Zeitschriften ist möglich. - Dieser Zugang muss von der Verwaltung zur Verfügung gestellt werden und nicht von einem Universitäts-Login  ii. Zum Beispiel Medline, Embase, PsycInfo, etc. als relevant  iii. Eine Bibliothek, die Zugang zu einer Reihe von Ressourcen bietet, nicht lediglich ein gemeinsam genutztes Ablagesystem, Forschungsergebnisse sind über eine elektronische/online Datenbank einfach und schnell verfügbar sind (ähnlich wie eine Universitätsbibliothek funktioniert)  iv. Ja, zu Endnote oder etwas Ähnlichem (einschliesslich Zugang, wenn darum gebeten wird).  Dies bezieht sich auf die Verwaltung, die den Zugang gewährt, nicht auf den Zugang über andere Mittel, z.B. die Universitätszugehörigkeit des Mitarbeiters. | i. Ja, einige Zeitschriften sind zugänglich, aber viele der benötigten Zeitschriften sind nicht zugänglich.  ii. Ja, Zugang zu einigen Datenbanken oder einer Datenbank, aber mehrere wichtige Datenbanken sind nicht verfügbar.  iii. Ja, aber es dauert lange, bis man Zugang zu Artikeln im Volltext erhält, oder man kann keinen Volltext erhalten, oder ja, aber viele der benötigten wichtigen Bücher sind nicht auf Lager.  iv. Hier gibt es keinen mittleren Punktewert, da nicht mehr als einen Ressource benötigt wird | i. Keine Abonnemente von Fachzeitschriften  ii. Keine Abonnemente von Datenbanken  iii. Kein Zugang zu einer Bibliothek oder elektronischen Bibliothek  iv. Keine Lizenzen für Referenzmanagement-Software |
| 12. Verfügt Ihre Verwaltung über definierte Prozesse zur Auftragsvergabe von Übersichtsarbeiten (Reviews) zu bestehenden Forschungsergebnissen? | Bereich 4: Verfügbarkeit von Hilfsmitteln und Instrumenten, die den Zugang zu Forschungsergebnissen und deren Anwendung erleichtern | Ja, es gibt einen standardisierten schriftlichen Ablauf, den die Mitarbeitenden bei der Vergabe von Forschungsaufträgen anwenden sollen.  Zu dieser Kategorie werden auch Rapid Review Prozesse gezählt. | Ja, aber die Methoden sind nicht verschriftlicht, ad hoc oder situationsspezifisch. | Es gibt keine Methoden, um Übersichtsarbeiten in Auftrag zu geben ODER die Verwaltung gibt keine Übersichtsarbeiten in Auftrag. |
| 13. Verfügt Ihre Verwaltung über Systeme zur Wissensverwaltung hinsichtlich Forschungsergebnissen?  Zum Beispiel. Systeme zum Abrufen, Zusammentragen, Speichern und Übersetzen von externen und internen Forschungsergebnissen | Bereich 4: Verfügbarkeit von Hilfsmitteln und Instrumenten, die den Zugang zu Forschungsergebnissen und deren Anwendung erleichtern | Es gibt gemeinsame Ablagesysteme, Datenbanken usw., die für die meisten relevanten Mitarbeiter leicht durchsuchbar und zugänglich sind.  Muss gut organisiert und strukturiert sein; nicht einfach nur ein grosser Ordner oder ein Laufwerk, auf dem die gesamte Palette von Dateien abgelegt sind (einschliesslich nicht-forschungsbezogener Dokumente) | Wird an einem Ort aufbewahrt und ist zugänglich, aber nicht indexiert oder leicht durchsuchbar. Die Verwaltung ist auf das Firmengedächtnis angewiesen, um zu wissen, welche Forschungsarbeiten durchgeführt wurden und wo sich diese befinden.  Zentralisiertes System, aber unorganisiert oder noch nicht vollständig entwickelt. | Es gibt keinen zentralen Speicherort und keinen Prozess zur Verwaltung von Wissen aus der Forschung. |
| 14. Hat Ihre Verwaltung in den letzten sechs Monaten interne Forschungsarbeiten zur Unterstützung der Politikgestaltung durchgeführt?  Zum Beispiel Fokusgruppen oder Zufriedenheitsumfragen. Diese Frage beinhaltet NICHT, ob die Verwaltung Evaluationen ihrer Politische Massnahmen und Programme durchgeführt hat. Dies wird in den Fragen 16-18 erfasst. | Bereich 5: Vorhandensein von Systemen und Methoden zur Generierung neuer Forschungsergebnisse für die Arbeit der Verwaltung | Muss in den letzten sechs Monaten und von Mitarbeitenden der Verwaltung durchgeführt worden sein. Umfasst mindestens eine grosse oder vertiefte interne Untersuchung oder mehrere kleinere interne Untersuchungen. | Eine kleine interne Untersuchung. | Nein, nie oder nicht in den letzten sechs Monaten. |
| 15. Hat Ihre Verwaltung in den letzten sechs Monaten externe Forschungsarbeiten zur Unterstützung der Politikgestaltung in Auftrag gegeben?  Gemeint ist externe Forschung zur Unterstützung der Politikgestaltung. Bei dieser Frage geht es nicht darum, ob die Verwaltung ihre politischen Massnahmen und Programme evaluiert. | Bereich 5: Vorhandensein von Systemen und Methoden zur Generierung neuer Forschungsergebnisse für die Arbeit der Verwaltung | Von einer anderen Organisation durchgeführte Forschungsarbeiten (möglicherweise in Partnerschaft mit dieser Organisation). Muss in den letzten sechs Monaten und mehr als einmal durchgeführt worden sein. | In den letzten sechs Monaten, aber nur einmal. | Nein, nie oder nicht in den letzten sechs Monaten. |
| 16. Fördert oder fordert Ihre Verwaltung die Integration von Evaluationen in die Planung und Entwicklung von politischen Massnahmen und Programmen?  *Die Fragen 16-18 schliessen extern in Auftrag gegebene Evaluationen mit ein.* | Bereich 6: Klare Methoden, welche angemessene und evidenzbasierte Evaluierungen der politischen Massnahmen und Programme ermöglichen | Es gibt eine explizit dokumentierte Anforderung der Verwaltung, dass die Evaluierung in jede Massnahme/jedes Programm eingebaut werden muss. | Ja, dies wird erwartet, aber nicht verlangt, oder es wird nicht von allen Programmen verlangt. | Evaluationen finden nicht oder nur gelegentlich statt, aber es besteht keine verwaltungsinterne Notwendigkeit, diese durchzuführen. |
| 17. Verfügt Ihre Verwaltung über dokumentierte Prozesse wie politische Massnahmen und Programme evaluiert werden sollen?  🡪 Falls *nein* weiter zu Frage 19 | Bereich 6: Klare Methoden, welche angemessene und evidenzbasierte Evaluierungen der politischen Massnahmen und Programme ermöglichen | Die Prozesse müssen im Detail darlegen, wie die Politische Massnahmen und Programme evaluiert werden sollen. | Ja, es gibt dokumentierte Prozesse, die sehr allgemein gehalten sind.  ODER dokumentierte Prozesse werden auf Fall-zu-Fall-Basis oder nach anfänglichen Vorbereitungen entwickelt | Keine dokumentierten Prozesse. |
| 18. Ermutigen oder erfordern diese Prozesse, Forschungsergebnisse in der Evaluation von politischen Massnahmen oder Programmen einzusetzen ODER basieren diese Evaluationsprozesse und -methoden auf Forschungsergebnissen?  Bezieht sich NICHT auf die Datenerhebung als Teil der Evaluation. Hier geht es darum, ob der von der Verwaltung angewandte Evaluationsansatz auf Forschungsergebnissen basiert, oder ob Mitarbeitende in der Gestaltung der Evaluation zur Nutzung von Forschungsergebnissen angehalten ist.  Wenn Frage 17 mit NEIN beantwortet wird, dann gibt es für diese Frage den Wert 1, auch wenn die Evaluation von einem Experten/einer Expertin durchgeführt wird. | Bereich 6: Klare Methoden, welche angemessene und evidenzbasierte Evaluierungen der politischen Massnahmen und Programme ermöglichen | Die Anforderung, Forschungsergebnisse zu nutzen, muss explizit und unmissverständlich sein.  (Dies kann sowohl die Recherche von Evaluierungsmethoden als auch von Szenarien umfassen. "Forschungsergebnisse" umfassen nicht lediglich Datenerhebung, darauf bezieht sich Frage 14.)  Entweder basieren die Prozesse auf Forschungsergebnissen, oder diese weisen Mitarbeitende dazu an, nach Forschungsergebnissen zu suchen, um ihren Evaluationsansatz zu unterstützen. | Die Prozesse beziehen sich auf Forschungsergebnisse, fördern oder fordern aber nicht, dass Forschungsergebnisse genutzt werden.  ODER die Evaluation wird von einem Experten durchgeführt, von dessen Ansatz angenommen werden kann, dass dieser durch Forschungsergebnisse beeinflusst wurde (eher indirekter Einsatz von Forschung) | Es gibt dokumentierte Prozesse, aber es besteht keine Forderung Forschungsergebnisse einzusetzen, oder es gibt keine dokumentierten Prozesse bezüglich der Evaluation. |
| 19. War Ihre Verwaltung in den letzten sechs Monaten auf Forschungsforen oder Konferenzen vertreten? | Bereich 7: Mechanismen, die zur Stärkung von Beziehungen zu Forschenden beitragen | Die Teilnahme an solchen Veranstaltungen war üblich und wurde von einer Reihe von Mitarbeitenden besucht. | Nur eine bestimmte Ebene von Entscheidungsträger/innen nimmt daran teil oder nimmt nur als eingeladene/r Referent/in teil oder nimmt nur selten teil. | Nein, nicht in den letzten sechs Monaten oder überhaupt nicht. |
| 20. Unterhält Ihre Verwaltung formelle, vertragliche Beziehungen zu externen Forschungseinrichtungen? | Bereich 7: Mechanismen, die zur Stärkung von Beziehungen zu Forschenden beitragen | Jede formell dokumentierte Beziehung zählt. Kurzzeitige Beziehungen sind in Ordnung, wenn diese zum Zeitpunkt des Interviews aktiv sind. Es müssen mehrere solcher Beziehungen bestehen, und es muss das Gefühl bestehen, dass diese (oder andere) wahrscheinlich weiter bestehen würden und dass das Bestehen solcher Beziehungen für ihre laufende Arbeit wichtig war. | Derzeit nur eine. | Nein, dies passiert nicht oder es gibt derzeit keine. |
| 21. Unterhält Ihre Verwaltung informelle, kooperative Beziehungen zu externen Forschungsorganisationen? | Bereich 7: Mechanismen, die zur Stärkung von Beziehungen zu Forschenden beitragen | Jede nicht formelle Beziehung (auch auf der Basis von Mitarbeitenden zu Mitarbeitenden) zählt hier. | Derzeit nur eine. | Nein, dies passiert nicht oder es gibt derzeit keine. |
| 22. Haben Mitarbeitende Ihrer Verwaltung Berufungen oder nebenberufliche Funktionen an Forschungsorganisationen? | Bereich 7: Mechanismen, die zur Stärkung von Beziehungen zu Forschenden beitragen | Übliche Beispiele hierfür sind Berufungen an Universitäten. Eine hohe Bewertung würde bedeuten, dass mehrere Mitarbeiter solche Positionen bekleiden. Dies gilt auch, wenn Mitarbeitende in Teilzeit bei der betreffenden Verwaltung arbeiten und auch bei einer Forschungsorganisation angestellt sind. | Derzeit nur eine. | Nein, dies passiert nicht oder es gibt derzeit keine. |
| 23. Haben externe Forschende in den letzten sechs Monaten in Ihrer Verwaltung an Beratungsgremien zur Politikgestaltung (oder ähnlichem) mitgewirkt? | Bereich 7: Mechanismen, die zur Stärkung von Beziehungen zu Forschenden beitragen | Die Beteiligung von Forschenden in diesen Rollen ist häufig, d.h. sie kommt mehr als einmal in sechs Monaten vor, und sie ist systematisch (nicht zufällig). | Nur einmal in den letzten sechs Monate. | Nein, dies geschieht nicht oder ist in den letzten sechs Monaten nicht geschehen. |
