# Supplementary material for: Exploring evidence use and capacity for health services management and planning in Swiss health administrations: A mixed-method interview study
Source: PLoS One. 2024 May 8;19(5):e0302864. doi: 10.1371/journal.pone.0302864 (PMC11078391; doi:10.1371/journal.pone.0302864)
Supplement: S2 Table — Exemplary quotes for codes of the category “relevancy of evidence in administration” of the final framework. (DOCX) [file pone.0302864.s004.docx]

**Supplementary information 3.** **Framework coding example.** Exemplary quotes for codes of the category “relevancy of evidence in administration” of the final framework.

| **Relevancy of evidence in administration (Category)** | |
| --- | --- |
| *Code* | *Quote* |
| Organizational culture | “We have in our department, I think, three or four PhD graduates, so we are all very … judgmental in the sense that this is something relevant to us. We also try to educate ourselves personally up to date.” |
| Cultural discrepancies between departments/units | “Within the administration, there are certainly administrative units that are a little more evidence-based than others. An office for environmental protection ... without evidence, nothing works there. Therefore, they do nothing without evidence. In the health sector, on the other hand, it is perhaps a little less ... we have different challenges.” |
| Focus of the management level | “The creed is that what is available or is accessible with a reasonable amount of effort. I have the expectation of my employees to obtain this data and incorporate it into their work.” |
| Self-conception of evidence-informed work | “We are extremely dependent on data. Evidence is something very important to us. It may not always be applied in a structured or systematic way. But ultimately, it is very often about asking ourselves the question, what is really happening, what are the facts?” |
| Pragmatism vs. evidence | “From a purely scientific point of view, you could increase the rescue times if, for example, you moved location A and location B for the rescue vehicles. But then in reality, you can't just ... it is not that easy to realize. It would need funds, you would have to invest, etc.” |
| Central evidence-base of work | “If it is about a specific area, then of course it [the engagement with evidence] can go extremely in-depth ... if we really have to plan a specific area, for example cardiac surgery or when commissioning services from hospitals ... [and] scientifically substantiate an overuse then it [the engagement with evidence] can go into great detail. In such situations, scientific publications from journals or reviews can be decisive. But that is rather rare.” |
| Health policy is generally not evidence-based | “For our canton, I have to say quite honestly, evidence rarely creates legitimacy, mostly it is the political will. Of course, if you want to do something, you need a data basis where you can show something, but I sometimes have the feeling that the political will is much more important, whether you find something opportune or not.” |
| Comparison to other cantons | “Sometimes it is simply a matter of comparing with the others; what do the others do? And then it's not necessarily evidence that decides, but "aha, the people in canton X do it that way too, well, so do the people in Y, so let's do it that way too". Of course, there is also a bit of this aspect. So what's the common sense or best practice, where you simply adopt certain things and does not ... review the numbers or do another study yourself.” |
