# Supplementary material for: Exploring evidence use and capacity for health services management and planning in Swiss health administrations: A mixed-method interview study
Source: PLoS One. 2024 May 8;19(5):e0302864. doi: 10.1371/journal.pone.0302864 (PMC11078391; doi:10.1371/journal.pone.0302864)
Supplement: S1 File — (DOCX) [file pone.0302864.s005.docx]

## S2 Guide. In-depth interview guide.

**Background**

1. ‎Can you briefly describe your current position and responsibilities?‎

*How long have you been in this position? ‎*

*How long have you worked in this administration? ‎*

**Role of Evidence**

1. ‎In making health policy, what information bases are relevant?‎

*Statistics such as hospital statistics?‎*

*How do you assess the availability of information and data for health policymaking?‎*

*How do you rate the linkage of data?‎*

1. ‎What do you think about the role of evidence in your work?‎

*What is the value of evidence in your work?‎*

*What evidence is helpful in your work?‎*

*In which areas of your work does evidence play a role, and where does it tend not to?‎*

*How do you rate the importance of evidence in health policymaking over the last ‎few years?‎*

1. ‎What similarities and differences in the role of evidence do you see between your administration’s various ‎areas of work (e.g., health care services vs. prevention)?‎

*What is the value of evidence in hospital planning compared to other fields?‎*

**Values regarding evidence**

1. ‎What are your general attitudes regarding the use of evidence in health policymaking?‎

**Engaging with evidence**

1. ‎How do you personally engage with evidence?‎

*How do you find and access evidence for your work?‎*

1. ‎What is the function of evidence in your work?‎

*In what situations is evidence particularly important?‎*

1. ‎How would you rate your knowledge and skills in using evidence?‎

*What enables you to deal with evidence (e.g., internal research, mandated ‎evaluation)?*

*How do you succeed in finding, assessing, and applying evidence?‎*

1. ‎What personal resources help you deal with evidence?‎

**Organizational factors**

1. ‎How would you describe the culture for using evidence in policy in ‎your administration?‎

*Are there incentives to use evidence? If so, what do they look like?‎*

*Is there an expectation that evidence should be used in health policymaking?‎*

1. ‎What are the attitudes of leaders in your administration toward using evidence in policymaking?‎

**Existing resources**

1. ‎How do you support your staff in using evidence?‎

*What resources and infrastructure does your administration provide?‎*

*Time, training, processes and systems, tools, access to experts, consulting mandates?‎*

**Needs**

1. ‎What are your needs for support in engaging with evidence in your work?‎

*Where do you expect more support?‎*

*Access, assessment, and application of research?‎*

*Potential for increased use of evidence?*

1. ‎Where do you see the potential for increased use of evidence in your administration?‎

*Where do you think it would be important to consult more evidence?*

1. ‎How could this potential be realized?‎

*What are the most important starting points to increase the value of evidence in your ‎ administration?‎*

**Existing collaboration with research**

1. ‎How would you describe the current collaboration with researchers and research institutions?‎

*With which institutions and individuals do you collaborate or exchange information?‎*

*Universities/universities of applied sciences, Obsan, auditing organizations, ‎evaluation offices?‎*

*What about the exchange between the cantons and the federal administration regarding ‎ evidence?‎*

**Examples**

1. ‎Can you give a concrete example where evidence was not ‎used, although it was presumably available?‎
2. ‎Can you give a concrete example of where evidence was used?‎

**Conclusion**

1. ‎Is there anything else you would like to add to this topic? Was anything left out? Do you ‎have any questions?‎

***Sociodemographic information***

- ‎Sex
- ‎Age
- ‎What is your highest tertiary level of education (university, college, college of education, or ‎arts)? None/Bachelor’s/Master’s/Doctorate degree
- ‎Have you completed any advanced/continuing studies at a university (e.g., continuing ‎education master’s MPH, MHA, MAS, MBA, or similar) or individual courses/modules of ‎these programs? (Title and subject/area)‎
- ‎Years of work experience in science/research
- ‎Management function yes/no; if yes, responsible for how many persons
